# Altered serum and cerebrospinal fluid TNF-α, caspase 3, and IL 1β in COVID-19 disease

**DOI:** 10.22088/cjim.13.0.264

**Published:** 2022

**Authors:** Mahtab Ramezani, Omidvar Rezaei, Ilad Alavi Darzam, Mohammadreza Hajiesmaili, Mahdi Amirdosara, Leila Simani, Abbas Aliaghaei

**Affiliations:** 1 Skull Base Research Center, Loghman Hakim Hospital, Shahid Beheshti University of Medical Sciences, Tehran, Iran; 2 Department of Infectious Diseases, Loghman Hakim Hospital, Shahid Beheshti University of Medical Sciences, Tehran, Iran; 3 Anesthesiology Research Center, Loghman Hakim Medical Center, Shahid Beheshti University of Medical Sciences, Tehran, Iran; 4 Neuroscience Lab, Anatomy and Cell Biology Department, School of Medicine, Shahid Beheshti University of Medical Sciences, Tehran, Iran

**Keywords:** Apoptosis, Inflammation, COVID-19

## Abstract

**Background:**

We evaluated the levels of the tumor necrosis factor-α (TNF-α), interleukin-1β (IL-1β), and caspase-3 in the cerebrospinal fluid (CSF) and serum of COVID-19 patients to improve our knowledge about underlying mechanisms caused by this virus in central nervous system involvement.

**Case Presentation:**

This case series study included six COVID-19 patients from March 26, 2020, to April 17, 2020, and six healthy control patients. CSF and serum levels of TNF-α, IL-1β, and caspase-3 have been assayed using monoclonal antibodies-based ELISAs.

Patients with COVID-19 had significantly higher level of IL-1β, TNF-α, and caspase-3 in serum (239.16±35.73 pg/ml, 100.50±12.49 pg/ml, 3.58±0.11pg/ml, p < 0.001) and CSF (146.66±17.55 pg/ml, 63.16±14.68 pg/ml,3.22±0.03pg/ml, p<0.001), respectively as compared to control. In addition, our results showed that these biomarkers were significantly higher in serum compared with CSF of the COVID-19 patients (p<0.001).

**Conclusion:**

This study provides essential information for understanding the pathogenesis of COVID-19 infection and sheds light on the potential mechanisms of virus transmission. The obtained data could be useful for designing new prevention and treatment strategies for COVID-19.

Coronavirus disease 2019 (COVID-19) is one of the main viruses that are primarily targeting the human respiratory system, but it can also spread to the central nervous system (CNS) from the respiratory tract. Patients with severe disease are highly likely to have CNS involvement and neurological manifestations (1, 2). In COVID-, inflammatory reactions play a critical role in pathogenesis, and the CNS and the immune system are complex systems that interact with each other (3). The inflammatory cytokine storm increases the severity of COVID-19 (4, 5). High blood levels of cytokines and chemokines have been detected in patients with COVID-19 infection, including IL1-β, IL-6, TNFα, etc. (6). Elevated levels of IL-1β and TNF-α in peripheral blood are of particular concern because neuroinflammation has been associated with both cytokines (7). It has been discovered that cytokines can cross the blood-brain barrier through a saturable transport mechanism (8). Caspases, as a group of intracellular cysteine proteases, have been shown to play a pivotal role in the regulation and execution of apoptosis. Within this group, caspase-3 is the main executioner protease, and its activation marks a point-of-no-return in the complicated cascade of apoptosis induction (9). The presence of activated caspase-3 in CSF is possibly caused by secondary insults due to necrosis of neurons. (10). TNF receptor superfamily activation of a surface death receptor initiates apoptosis, which activates caspase-3. As well, cytokines such as interleukin IL-1β and IL-6 could trigger apoptosis in cells; in this way, the mitochondria releases cytochrome c, which activates caspase 3 (10).

Most coronaviruses share common viral structures and pathways of infection; thus, the identified pathomechanisms for other coronaviruses may also be observed for SARS‐CoV2. Recent evidence has suggested that neuro-invasion and neurotropism is a common feature of human coronaviruses (2). In the reported patients with SARS-CoV1, CSF was positive for the virus (11). However, recent studies of CSF in patients with COVID-19 have shown contradictory results (12, 13). The CSF parameters have only been analyzed in a limited number of patients (14, 15). To date, however, caspase-3 status in COVID-19 patients has not been studied. Hence, we aimed to determine the CSF and serum levels of caspase-3 along with inflammatory markers; TNF-α and IL-1β in patients with COVID-19 and compare it with healthy control patients. We also utilize paired serum and CSF samples in patients with COVID-19 to compare systemic and CNS inflammatory responses. This study results improve our knowledge about the underlying mechanisms of COVID-19 that can be reflected by molecular changes in this disorder.

## Case presentation

This study was performed on six patients with COVID-19 and six age and sex-matched healthy control group. Patients consisted of those admitted in the ICU of a university-affiliated major hospital between March 26, 2020 - the beginning of the Iran epidemic – and April 17, 2020. Six patients with COVID-19 compatible respiratory symptoms intubated due to respiratory distress and altered consciousness range from drowsiness to confusion in the first three days were enrolled. Patients with loss of consciousness secondary to sepsis, focal neurological deficit, hypoxia, and patients with neurological signs and symptoms (including headache, vertigo, anosmia or hyposmia, and ageusia or hypogeusia) at onset were excluded. 

A spiral chest CT scan without contrast confirmed COVID-19; also, all the patients tested positive for SARS-CoV-2 (by 2019 Novel Coronavirus Real-Time RT-PCR) on the second day of admission. As a control, we included the CSF and serum of six patients requiring spinal anesthesia for elective orthopedic surgery. Venous blood and CSF samples were taken simultaneously from patients with COVID-19 within five days following test positive for SARS-CoV-2 and randomly from the control group. Analysis of CSF, including the leukocyte count, protein, lactate, and sugar levels, along with COVID-19 PCR, were performed. The excess of CSF was stored in 1ml aliquot at -20°C. Five ml of Venus blood samples were collected from all these patients. Subsequently, the samples were centrifuged at 4000 g/ 10 min, and the upper supernatant fluid was separated. All CSF and blood samples were analyzed blinded in clinical information by trained biochemists and run-in duplicate. The enzyme-linked immunosorbent assay (ELISA) was conducted on CSF and blood samples to assess the levels of IL-1β (Abcam, ab46052), TNF-α (Abcam, ab181421), and caspase-3 (Abcam, ab220655). All statistical analyses were performed using SSPS Version 22.0 (SPSS, Inc., Chicago, IL, USA). We showed qualitative data and quantitative data by mean ± standard deviation (SD) and frequency, respectively. In addition, categorical data using chi-square and Fisher's Exact test were analyzed. The independent t-test was performed to assess comparisons of numeric values. Correlation between CSF and serum levels of caspase-3, TNF-α, and IL-1β were performed using the Pearson correlation test. We calculated adjusted mean values in both studied groups using one-way ANCOVA (covariance analysis) to control covariates. The continuous variables such as spinal anesthesia and comorbidity were considered as the covariate in the variance analysis. The level of statistical significance was set at p < 0.05.

This study was performed on six patients with COVID-19 and six age and sex-matched healthy control group (P=1.0, P=0.877, respectively). Moreover, there were no notable differences in CSF routine analysis and comorbidity between the two groups **(**table 1). 

In all six patients, RT-PCR assays of the CSF samples were negative for SARS-CoV-2. The CSF and serum levels of IL-1β, TNF-α, and caspase-3 increased in all patients in comparison to control (table 2). The levels of IL-1β, TNF-α, and caspase-3 were significantly higher in serum compared with the CSF in patients with COVID-19 (table 3). Furthermore, CSF TNF-α and caspase-3 concentrations were positively correlated with serum concentrations (r = 0.821, P =0.044; r=0.991, P=0.001), respectively. We conducted ANCOVA to determine the possible confounding effects of spinal anesthesia and comorbidity covariates. This analysis demonstrated similar results, even after considering the covariates. It means that the observed significant difference between the study group and levels of TNF-α, IL-1β, and caspase-3 was not attenuated after adjusting for anesthesia and comorbidity of study subjects. 

**Table 1:** Demographic and clinical characteristics of subjects in the study groups

**Variables **	**Control**	**Case**	**P-value**
Sex (N) Male/female	4/2	4/2	- 1.00
Comorbidity Yes No	- 6	2 4	0.455
Spinal anesthesia Yes No	6 0	0 6	0.002^
Age (Mean±SD)^$^	53.33±19.73	51.66±20.81	0.877
CSF analysis Appearance Color RBC WBC Protein Sugar Lactate LDH	Clear Non 0 0 76.33±20.63 84.75±31.51 11.25±1.50 33.50±5.44	Clear Non 0 0 58.00±17.46 77.16±16.91 10.0±0.00 35.12±0.00	- - - 0 0.128 0.606 0.153 0.663
RT-PCR for SARS-CoV-2 in CSF	Negative	NA^#^	-

^^^ P<0.05;^ $^ Standard deviation; ^#^Not available

**Table 2:** The mean ± SD of inflammatory and apoptosis markers in study groups

**Biomarkers **	**CSF** **Case vs. Control**	**Serum** **Case vs. Control**	**P-value**
IL-1β(pg/ml)	146.66±17.55 vs. 46.66±15.70	239.16±35.73 vs. 49.83±23.81	0.001
TNF-α(pg/ml)	63.16±14.68 vs. 23.33±6.77	100.50±12.49 vs. 29.33±17.04	0.001
Caspase-3(pg/ml)	3.22±0.03 vs.0.15±0.04	3.58±0.11vs. 0.14±0.03	0.001

^ P<0.001

**Table 3:** The mean ± SD of inflammatory and apoptosis markers in patients with COVID-19

**Biomarker **	**CSF**	**Serum**	**P-value**
IL-1β(pg/ml) TNF-α(pg/ml) Caspase-3(pg/ml)	146.66±17.55 63.16±14.68 3.22±0.03	239.16±35.73 100.50±12.49 3.58±0.11	0.001^^^ 0.001 0.001

^^^P<0.001

## Discussion

In our case series, ARDS due to SARS-CoV-2 infection was correlated with increased CSF and serum levels of caspase-3, IL-1β, and TNF-α. The CSF routine analysis in our patients was within normal limits and negative for SARS-CoV-2; therefore, COVID-19 seems not to cross the blood-brain barrier (BBB), consistent with the previous study (13). A series of case reports have reported that COVID-19 could present with headache, altered mental status, acute CVD, acute necrotizing hemorrhagic encephalopathy, and acute Guillain–Barré syndrome; however, all the CSF routine analysis was within a normal range, suggesting that SARS-CoV-2 neither cross the BBB nor cause meningitis or encephalitis (16-20). A recent study of the brain pathological findings in COVID-19 patients with cerebral infarction have shown only nonspecific brain edema and atrophy without any signs of direct infection (21). Wan et al. found that the cytokine storm is crucial to the progression of COVID-19 and can lead to severe complications and death (22). In an isolated report, CSF analysis also showed markedly elevated pro-inflammatory cytokines (23).

The prior evidence showed the effects of systemic inflammation on BBB function in animals and humans' healthy and diseased brains (24). Non-disruptive changes in BBB during systemic inflammation can also promote pathogen neuroinvasion. The BBB serves as a signaling intermediate by increasing the production of pro-inflammatory cytokines such as TNF-α and IL-1β in response to systemic stimuli and the blood-to-brain transport of TNF-α through up-regulation of receptor-mediated transcytosis, which then acts on the brain.

 By the time COVID-19 infection involves the respiratory tract, it can cause a wide range of symptoms, from mild to severe, resulting in releasing pro-inflammatory cytokines, such as interleukin IL-1β TNF-α (24). Since COVID-19 attaches to the toll-like receptor, pro-IL-1β and TNF-α are released. Then, active mature IL-1β and TNF-α can trigger a fever, pulmonary inflammation, and fibrosis. Interestingly, it has been shown that they may have therapeutic effects on many inflammatory diseases, such as viral infections if pro-inflammatory IL-1 family members are suppressed (25, 26). 

As mentioned above, apoptotic cell death mainly occurs through two separate pathways, TNF receptor superfamily and cytokines activation, which activates caspase-3 (10). These findings indicate that apoptosis and inflammatory processes were simultaneous in the CSF and serum. Since pro-inflammatory cytokines, including TNF-α and IL-1β, can cross the blood-brain barrier via active transport mechanisms, neuro-inflammatory responses can be developed from systemic sources (8). 

Therefore, the significantly elevated levels of IL-1β, TNF-α, and caspase-3 in serum, along with modest or increased levels in CSF of the COVID-19 patients, support the concept that systemically-derived inflammatory responses can mediate CNS neuroinflammation during the acute phase of the disease.In identifying the abnormalities in cranial nerves, brain parenchyma, and olfactory bulb of the COVID-19 patients, neuropathology and CNS imaging play a crucial role (27). Several patients have reported sudden hyposmia and anosmia as concomitant symptoms of COVID-19 infection over the last few months. Since these patients have not been explicitly recognized for anosmia or hyposmia, COVID-19 can not be diagnosed clearly (26).

This study analyzed combined, fresh frozen serum and CSF samples concurrently and showed that in COVID-19, systemic and CNS inflammatory indices are simultaneously altered. The final consideration is the degree to which the systemic (serum) and neuro-inflammatory (CSF) responses were consistent. Concordant responses in which the levels of pro-inflammatory cytokine and apoptosis in COVID-19 were significantly higher in serum than CSF would support the theory that neuroinflammation is driven by systemic inflammation. 

These findings are important because it seems that CNS involvement in COVID-19 is at the molecular level. Probably when the inflammatory marker levels reach the CNS threshold, neurological manifestations could present. Following up, the COVID-19 patients for mentioned symptoms can illuminate the virus' remote effect in the CNS. The role of the BBB in containing the virus and preventing it from gaining access to the neural tissues needs to be further explored in patients diagnosed with COVID-19. 

In our study, the sample size was small, and the findings should be interpreted with caution. As a result of limited financial resources, we could not use the maximum sample size and other biomarkers' measurements. However, future investigation is recommended in larger sample sizes and concomitant measurement of other biomarkers.

In conclusion, this study shows that COVID-19 might be involved in the CNS due to a molecular basis. It also sheds light on the potential mechanisms of virus transmission and provides insights into the pathogenesis of COVID-19 infection.
